# Primary sensory map formations reflect unique needs and molecular cues specific to each sensory system

**DOI:** 10.12688/f1000research.17717.1

**Published:** 2019-03-27

**Authors:** Bernd Fritzsch, Karen L Elliott, Gabriela Pavlinkova

**Affiliations:** 1 Department of Biology, University of Iowa, Iowa City, USA; 2 Institute of Biotechnology of the Czech Academy of Sciences, Vestec, Czech Republic

**Keywords:** primary sensory maps, retinotopic map, olfactory map, cochleotopic map, vestibular map, taste map

## Abstract

Interaction with the world around us requires extracting meaningful signals to guide behavior. Each of the six mammalian senses (olfaction, vision, somatosensation, hearing, balance, and taste) has a unique primary map that extracts sense-specific information. Sensory systems in the periphery and their target neurons in the central nervous system develop independently and must develop specific connections for proper sensory processing. In addition, the regulation of sensory map formation is independent of and prior to central target neuronal development in several maps. This review provides an overview of the current level of understanding of primary map formation of the six mammalian senses. Cell cycle exit, combined with incompletely understood molecules and their regulation, provides chemoaffinity-mediated primary maps that are further refined by activity. The interplay between cell cycle exit, molecular guidance, and activity-mediated refinement is the basis of dominance stripes after redundant organ transplantations in the visual and balance system. A more advanced level of understanding of primary map formation could benefit ongoing restoration attempts of impaired senses by guiding proper functional connection formations of restored sensory organs with their central nervous system targets.

## Introduction

Sensory organs are the windows of the brain to the environment, permitting behavioral interactions with conspecifics, prey, and predators. Finding an appropriate mate, being able to avoid being eaten, and finding food are essential features for survival and propagation. Sensory organs filter out the appropriate information for these tasks and relay it to the brain to elicit adequate motor responses ^1– 3^. Sensory map features depend on the specific sensory modality and the relevant information to be extracted. For example, somatotopic maps project a topographic array of sensors to reflect the sensor distribution, density, and activity of the skin to the brain ^4– 6^. Similarly, the retinotopic map projects distinct areas of the retina and the corresponding visual field as a two-dimensional (2D) map to the target brain area ^7, 8^, whereas the cochlea map projects a unidimensional map of distinct frequencies to specific areas of the cochlear nuclei ^9^ and auditory cortex ^10, 11^. Beyond primary sensory maps, central map formation underlies binocular vision and depth perception ^12, 13^. Likewise, the auditory space map is generated through binaural interactions ^14– 16^ whereas the mechanosensory lateral line ^17^ and the electrosensory space map ^18^ are generated through integration of distributed sensors across the body. In contrast to these emerging centrally synthesized maps and continuous primary maps, discrete olfactory maps project unique properties of the odorant stimuli perceived by distributed olfactory neurosensory cells convergently onto specific glomeruli ^10, 19, 20^. A variation of the latter theme is the incomplete segregation of movement detection in the vestibular system, where angular movements always cause concomitant linear acceleration. This causes partial convergence of afferents from organs dedicated to either linear or angular acceleration perception ^21, 22^. Even more difficult to understand are maps where a given stimulus and its intensity are differentially coded as the tastants for the yet-to-be-fully-defined taste map ^23– 26^.

During the last century, specific properties of a given sensory map and basic rules how to form them, such as the chemoaffinity theory ^27^ and activity-mediated synaptic plasticity theory ^28, 29^, have been worked out for some primary maps. Understanding the molecular cues that guide primary map development, the plasticity of primary map development mediated by activity to sharpen the map in neonates and adults ^30– 33^, and the translation of primary sensory afferent map formation into cortical and midbrain maps for multisensory integration ^2, 6, 34, 35^ will be the defining achievements of the 21st century. Toward this end, we provide here an overview of various primary sensory maps of mammals, characterized by continuous and discrete map properties ^33^. All primary maps require that a peripheral sense be wired to independently developing central target neurons by molecular cues in the target and matching cues expressed in the neurons as they navigate toward their target. Our aim is to turn primary sensory map formation into a neuronal pathfinding problem that combines with cell cycle exit to generate an embryonic primary map for each sense. Uncovering regulatory aspects of map formation across senses will facilitate sensory restoration badly needed for sensory repair of seniors in our rapidly aging societies.

## Primary sensory maps compared

The six primary sensory maps of mammals have unique features and seemingly use distinct molecular cues, cell cycle exit, and activity combinations during development, regeneration, and plasticity. We will start with the best molecularly understood map formations followed by the less well understood map formations in the hindbrain, ending with the least understood map for taste that has recently seen dramatic revisions from past insights ^24, 36^.

## Molecular odorant map

### Adult map organization

**Figure 1.**
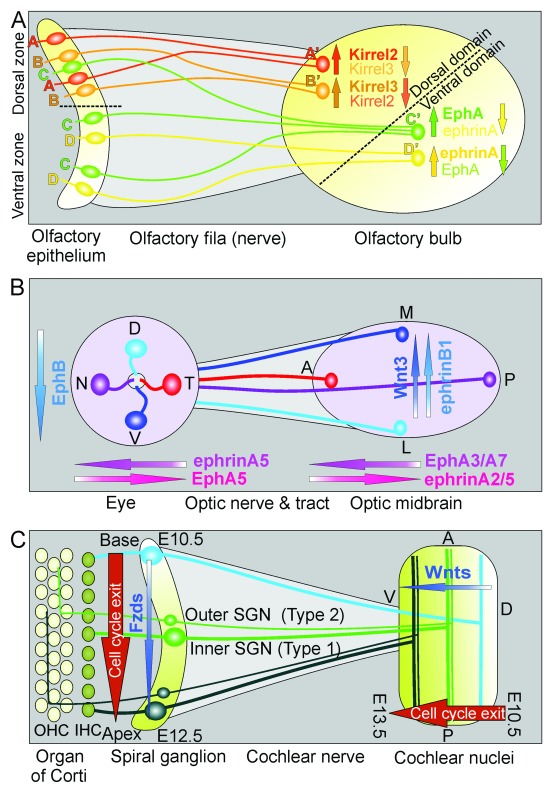
Development of three distinct mammalian sensory maps. Molecular cues ( **A, B**) and spatio-temporal cues ( **C**) are shown for the nearly non-spatial olfactory map ( **A**), the two-dimensional (2D) retino-tectal map ( **B**), and the unidimensional auditory map ( **C**). ( **A**) The olfactory map defines different olfactory receptor molecules in the dorsal and ventral zone of the olfactory epithelium. Receptor cells displaying distinct olfactory receptors ( **A**– **D**) project their axons to the dorsal and ventral domain of the olfactory bulb where they converge and initiate olfactory glomeruli formation. Note that olfactory fibers sort before they reach the olfactory bulb and that some ventral zone receptors are expressed in the dorsal zone but afferents sort to the ventral domain. Different opposing gradients of receptors facilitate further the sorting of olfactory afferents. Within this limited topology, the distribution of specific olfactory receptor–expressing receptor cells is fairly random. ( **B**) The retino-tectal system maps a 2D surface (the retina ganglion cells) onto another 2D surface (the midbrain roof or tectum opticum) via highly ordered optic nerve/tract fiber pathways. Within the midbrain, the presorted fibers are further guided by molecular gradients matching retinal gradients of ligand/receptor distributions. ( **C**) The auditory map is unidimensional, projecting a species-specific frequency range from the mammalian hearing organ, the organ of Corti via orderly distributed spiral ganglion neurons (SGNs), and their fibers in the auditory (cochlear) nerve onto the ventral cochlear nucleus complex. Both SGNs and cochlear nucleus neurons show a matching temporal progression of cell cycle exit followed by matching differentiation that could be assisted by spatio-temporal expression changes of receptors and ligands (shown here are the putative Wnt/Fzd combinations) that further support the fiber sorting. Note that this map projects a single frequency of an inner hair cell of the organ of Corti via a set of SGNs onto longitudinal columns of cochlear nucleus neurons in a cell-to-band projection and thus is not a point-to-point map as the olfactory and visual map. Moreover, afferents innervating multiple outer hair cells (OHCs) generate a band-to-band projection centrally. A, anterior; D, dorsal; L, lateral; M, medial; N, nasal; P, posterior; T, temporal; V, ventral. Modified after 12, 41, 53– 58.

Since the cloning of genes encoding a family of odorant receptor (OR) nearly 30 years ago ^37^, the understanding of olfactory map formation has leapfrogged to be perhaps the best molecularly understood sensory map. The basic principle is that a given olfactory sensory neuron (OSN), coding for a given OR, projects its axon to a molecularly specified olfactory glomerulus in the olfactory bulb (OB), where it converges with axons of other OSNs coding for the same OR ^20, 38– 40^. Thus, OSNs coding for the same OR converge to the same glomerulus ( Figure 1A). In the mouse, this results in a discrete expression of one of about 1100 ORs in a given OSN whose axon converges onto one or few of the roughly 3600 glomeruli. OR expression is not completely random but splits the olfactory epithelium into major divisions along the dorso-ventral axis, each with medio-lateral bands of randomly distributed OSNs that project to dorso-ventrally distinct sets of olfactory glomeruli ^38, 39, 41^. Specific odorant information is thus perceived by OSNs within certain zones that are, however, nearly randomly distributed within these zones. This is particularly obvious in mammals with a reduced complement of olfactory receptor genes that form glomeruli only in the ventral part of the OB ^42^. Odor information is encoded in the odorant-specific glomeruli and not in the topology of OSNs in the olfactory epithelium. This organizational principle allows OSNs to be continuously replaced ^43^ without any change in the important central information storage ^1, 34^. The brain learns and recognizes patterns of glomerular activity elicited by different odors ^44^.

### Development

**Figure 2.**
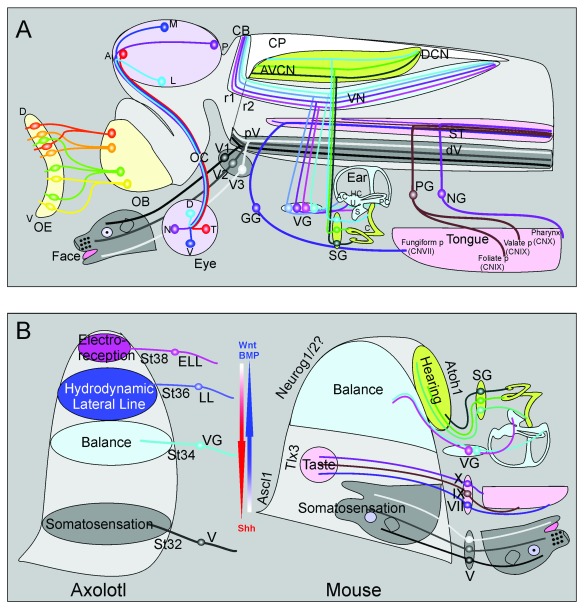
Distribution of sensory maps and the development of hindbrain sensory maps. ( **A**) Schematic presentation of the main features of the six cranial senses projected onto an embryonic mouse brain. (Pale yellow, left) Distributed olfactory sensory neurons of the olfactory epithelia coalesce their axons before reaching a specific olfactory glomerulus in the olfactory bulb (OB). (Pale lavender) Axons of retina ganglion neurons leave the eye orderly to project via the optic nerve to the optic chiasm (OC). Crossed contralateral axons form the orderly optic tract that distributes axons within the midbrain using matching gradients of several factors. (Gray) The trigeminal ganglion has three distinct branches and matching sensory neuron populations that reach different areas of the face. The central axons form in a temporal progression resulting in an inverted presentation of the face. (Pale pink) Taste buds of the tongue and pharynx are innervated by three cranial nerves that form a somewhat orotopic central projection to the solitary tract. (Light blue) The five vestibular sensory organs are innervated by somewhat orderly distributed sensory neurons that project via the vestibular nerve. Within the brain, vestibular afferents from different ear organs are partially segregated and partially overlapping in the various vestibular nuclei as well as the posterior lobes of the cerebellum. (Pale green) The organ of Corti of the cochlea is innervated by a temporally generated longitudinal array of spiral ganglion neurons that project in an orderly organization to dorso-ventral distinct regions of the cochlear nucleus complex, projecting a one-dimensional frequency array along the cochlea onto a matching frequency array of afferents in the cochlear nuclei. ( **B**) (Left) In the axolotl, there is a timing factor of afferent ingrowth such that the most ventral trigeminal projection reaches the hindbrain first (V at stage 32) whereas the most dorsal projection from the electroreceptive (lateral line) ampullary organs reaches the most dorsal part of the hindbrain last (ELL, stage 38). The inner ear vestibular ganglia (VG, stage 34) and mechanosensory lateral line ganglia (LL, stage 36) are reaching the alar plate between those extremes. (Right) In the mouse, the dorso-ventral patterning of the hindbrain is driven by countergradients of Wnt/BMP and Shh to regulate expression of transcription factors defining various nuclei. How these gradients define the positon of central nuclei and afferents is not completely clear. A temporal gradient of afferent development and projection development has thus far been demonstrated only for the spiral ganglion, taste and trigeminal system where the first neurons to form are the first to project to the most ventral part of their respective tract. Note that the auditory nuclei show an apparent inversion such that the most ventral projection from the basal spiral ganglion ends up in the more dorso-medial part of the cochlear nuclei because of the morphogenetic changes in cochlear nucleus neuron position. A, anterior; AC, anterior crista; Ascl1/Mash1, achaete-scute family basic helix-loop-helix transcription factor 1; Atoh7, atonal basic helix-loop-helix transcription factor 7; AVCN, antero-ventral cochlear nucleus; BMP, bone morphogenic protein; C, cochlea; CB, cerebellum; CN V, VII, IX, X, cranial nerve V, VII, IX, X; CP, choroid plexus of IV ventricle; D, dorsal; DCN, dorsal cochlear nucleus; dV, descending trigeminal tract; ELL, electroreceptive (ampullary organ) lateral line; GG, geniculate ganglion; HC, horizontal crista; L, lateral; LL, (mechanosensory) lateral line; M, medial; N, nasal; Neurog1/2, Neurogenin 1/2; NG, nodose ganglion; OB, olfactory bulb; OC, optic chiasm; OE, olfactory epithelium; P, posterior; PC, posterior crista; PG, petrosal ganglion; pV, principal trigeminal nucleus; r1, rhombomere 1; r2, rhombomere 2; S, saccule; SG, spiral ganglion; Shh, sonic hedgehog; ST, solitary tract; T, temporal; TIx3, T-cell leukemia homeobox 3; U, utricle; V, ventral; V1, ophthalmic branch of trigeminal nerve; V2, maxillary branch; V3, mandibular branch; VG, vestibular ganglion; VN, vestibular nucleus complex. Modified after 5, 12, 17, 33, 36, 54, 56, 63– 66.

The main and accessory (vomeronasal) olfactory epithelium develops from the olfactory placode that also gives rise to a set of gonadotrophin-releasing hormone (GnRH)-positive neurons migrating into the hypothalamus ^45– 48^. This allows the olfactory system to interconnect with the retina in some species ^49^. A sequence of basic helix-loop-helix (bHLH) genes, in combination with other transcription factors, guides the transformation of the olfactory placode cells into OSNs ^50, 51^. Dorso-ventral zones of ORs are expressed in selective OSNs and each OSN projects specific odorant molecule information to a given glomerulus ^52^. Matching gradients of OR expression define the pathfinding properties of OSNs to select a given band of glomeruli and a specific glomerulus within that band ( Figure 1 and Figure 2). Misexpression of a given OR in another set of OSNs results in misdirection to a different glomerulus. This supports the idea that both the selection of a given OR and the level of gene expression bestow on an OSN an identity that allows the OSN growth cone to navigate to a specific glomerulus. The G protein–coupled ORs define expression levels of adenylate cyclases such as *Ac3* ^33^. Knockouts of *Ac3* lead to disorganized OR projections. This appears to be related to *Ac3*-mediated activation of downstream guidance cues via cAMP/CREB/PKA, such as neuropilin 1 ( *Nrp1*). Gradients of *Nrp1* code for anterior-posterior patterning ^53^ in combination with matching expression of semaphorins ^59– 61^. However, detailed tests question the proposed model of *Nrp1* guidance by showing more complicated outcomes inconsistent with the simple *Nrp1* gradient model ^54^. Since G-coupled ORs are found on the growth cone of OSNs, those ORs could locally interact with the environment to guide confined responses via the cAMP/PKA intracellular signal cascades. Though clearly important, a gradation of G protein/cAMP alone is not the only cue, and *Robo/Slit* is used for larger-scale dorso-ventral patterning ^40, 62^. In addition, two different classes of OSNs have been identified and their axons sort out as they extend toward the OB, leading to a complete segregation of axons of dorsal but not ventral OSNs ^40^. This fiber sorting ( Figure 1A and Figure 2A) happens prior to and even in the absence of OBs, establishing a topographic order of OSN axons as they approach the OB ^53^. Further refinement of the olfactory mapping is achieved through differential expression and activity-regulated levels of *ephrinA* ligands and *Eph-A5* receptors as well as the molecularly related *Kirrel2/3* ( Figure 1A). In a given glomerulus, there is an opposing gradient of either the *Kirrel2/3* pair dorsal or *ephrinA/EphA5* ventral. This expression defines a dorsal and a ventral domain of glomeruli ( Figure 1A) matching to the dorso-ventral zones of OR expressing OSNs in the olfactory epithelium. Thus, although the dorso-ventral patterning of bands of OSNs to project to bands of olfactory glomeruli seems to be settled, the details of antero-posterior patterning remain less clear and seemingly are less precise ^54^.

### Olfactory epithelium manipulation

Past work has established that ingrowing OSN axons of transplanted olfactory epithelia can generate glomeruli wherever they project to in the forebrain or midbrain ^45^ but are unable to form glomeruli in the hindbrain ^67^. These placode transplantation experiments suggest that perhaps OSNs play a role in sculpting their own target area in the forebrain and midbrain. OB formation depends on ingrowing OSN axons, and no OB forms in mammals without an olfactory epithelium ^42, 68^. This indicates a self-organizational principle of OSNs beyond fiber fasciculation ^53^ that requires additional molecular exploration to help restore smelling to anosmic people.

## Retinotopic map

### Adult organization

Retinal ganglion neurons (RGNs) in a given position are driven by local spontaneous or induced activities in their specific receptive field of visual stimuli. RGNs send this information through their terminals onto a matching position of the roof of the midbrain, known as the non-mammalian optic tectum or mammalian superior colliculus ( Figure 1B and Figure 2A). Sperry’s ^27, 69^ experiments on frogs showed that a severed optic nerve re-establishes a functional map. However, rotating the eye before RGNs re-establish midbrain connections results in mismapping of the visual field that cannot be corrected for by activity. Sperry therefore proposed a chemoaffinity map that guides neurites from specific areas of the visual field/retina to matching positions of the midbrain. This basic idea led to a mathematical model of molecular countergradients ^70^. Sperry’s experiments and Gierer’s model stimulated the discovery of an orthogonal diffusion gradient of ephrin ligands and receptors in the retina and matching expression in the midbrain ^13, 33^. These gradients ensure that a given ganglion cell projects to a matching area of the midbrain ( Figure 1B). The retinotopic map projects a continuous topographic set of visual field information encoded by RGNs from one surface (the retina) point-to-point onto another surface (the midbrain) for further processing of the 2D topological information. This helps, for example, direct attention to specific objects, in particular moving objects, as is obvious after visual cortex lesions, known as “blindsight” ^71, 72^.

### Development

The retina develops as an evagination of the diencephalon that interacts with the lens placode for normal eye development ^46, 73^. Blocking retina evagination results in cellular transformation of the diencephalic wall with distinct retinal receptors and various retinal neuron layers ^74^. In contrast to the olfactory epithelium and its continuous renewal of OSNs, all retina neurons and sensory cells develop once in a concentric progression ^75^ through clonal expansion out of proliferative precursors using a series of transcription factors that define, in combination, specific retinal cell types ^76^. RGNs require the bHLH factor *Atoh7* and other factors for their differentiation ^77– 79^ to form the roughly 30 recognizable RGN types ^80^. How *Atoh7* and downstream transcription factors regulate the molecular guidance cues that allow a given RGN to exit the retina ^80^, sort along the optic nerve ^81^, and grow through the optic chiasm ^82^ to project, via the orderly optic tract ^83^, to a discrete region of the contralateral midbrain ^80^ remains incompletely understood ^12^. Graded expression of several molecules and receptors redundantly defines how the surface of the retina is mapped via targeted projection of RGNs onto the midbrain ^12, 33^. *Ephrin-A/EphA* has naso-temporal and *ephrin-B/EphB* dorso-ventral concentration-dependent attractive and repellent effects that define a narrow region in which terminal arbors of a given RGN can form. Eliminating multiple ligand/receptor pairs causes broad distribution of RGN axons; however, some very crude topology remains even after the main ligand/receptor pairing has been deleted ^13^. Multigene knockouts combined with removal of activity result in diffuse and broad innervation ^8, 80^. Additional molecular gradients are provided by a *Wnt3* gradient that defines, redundant to the *ephrin-B/EphB* gradient, the medio-lateral slope in the midbrain for dorso-ventral RGN axonal sorting ^33, 84^. The midbrain *Wnt3* gradient is translated into differential projections using *Ryk* gradients on RGN axons to modify, via repellent actions, the attraction mediated by Fzd receptor activity. Additional redundancy is provided by other secreted factors like *En-2* ^85, 86^. Activity of axons is not needed to define the overall projection ^87, 88^, but axonal arbors in the midbrain become less confined without activity. If both molecular map and activity are disrupted in combined mutants, the resulting maps of individual RGNs can cover large areas of the midbrain ^89^. This demonstrates that neuronal activity combines with molecular specificity to sharpen the retinotopic map ^7^ and provides the basis for ocular stripe formation in three-eyed frogs ^90, 91^.

### Eye manipulation

Molecular cue interactions with activity-mediated refinement result in “ocular dominance” column formation after additional eye transplantations ^91^. Differential eye activity is needed for the formation of these “ocular dominance” stripes ^29, 90^. “Ocular dominance” stripes also form after crushing an optic nerve results in misguided regeneration. Such stripes are maintained only if the contralateral nerve is either eliminated or also regenerates ^92, 93^. In line with the role of patterned activity in these processes is the absence of RGN axon segregation in the bilaterally projecting retino-midbrain systems found in fossorial vertebrates ^94^. How activity relates to neurotrophic release and thus long-term sustenance of regenerated RGNs remains debated ^33, 80^. Beyond three-eyed frogs and optic nerve crush-mediated “ocular dominance” stripe formation, some transplantation studies claim successful regrowth of RGN axons from the spinal cord to the midbrain ^95^ and RGN axons apparently can innervate the olfactory cortex ^96, 97^ or can restore visual guidance even after transplantations to unusual positions on a tadpole ^98, 99^, indicating alternative ways for visual information flow to the brain. How far such effects in amphibians can be translated to mammalian optic nerve regeneration ^80^ remains to be seen.

## Somatosensory map

### Adult organization

The cortical somatosensory map is the prototypical surface-to-surface map whereby dermatomes are mapped onto the cortex ^100^, and local variations ^101^ reflect various sensor densities and functional differences ^102, 103^. The somatosensory map thus is a 2D surface-to-surface projection comparable to the retinotopic map. However, in contrast to the simple retina surface projecting onto the tectal surface, closer examination reveals a complicated relationship between primary somatosensory afferent input and the formation of distorted, continuous surface map in the spinal cord and brainstem and the somatosensory map in layer IV of the somatosensory cortex ^6, 104^. Manipulating the periphery affects the central map, but the details of how phantom sensations are generated or how maps are altered after peripheral manipulations remain somewhat obscure ^6, 105, 106^.

### Development

For simplicity, we concentrate here on the trigeminal somatosensory system and exclude the spinal cord somatosensory map formation. The trigeminal sensory system is composed of ganglion neurons with three distinct embryonic origins: the trigeminal ganglion derived from both trigeminal placode and neural crest ^5^ and the mesencephalic sensory neurons of the mesencephalic trigeminal nucleus (MesV) derived from the brain ^107^. Loss of *Npr2* results in lack of bifurcation, blocking MesV branches from leaving the brain and thus depriving the brain of proprioceptor input ^108^. Topology of trigeminal ganglion neurons is defined by diffusible factors ( *Wnt*, *Fgf8*, and *Bmp4*) and localized expression of various transcription factors ( *Tbx1*, *Onecut*, and *Hmx1*) as well as differential expression of neurotrophins *Ntf3* and *Bdnf* ^5^ that enable innervation of distinct regions of the facial skin. Projections into the hindbrain develop before peripheral processes reach the skin target ( Table 1), indicating that trigeminal central processes are guided independently of their peripheral targets. The trigeminal nucleus target neurons in the terminal nuclei depend on *Mash1/Acsl1* that is directed in its expression within the hindbrain dorso-ventral patterning mediated by BMP/Wnt/Shh gradients, as in the spinal cord ^109^. Gradients of these factors may also play a role in afferent guidance ^110– 112^ but details remain to be worked out. Trigeminal ganglion afferents entering rhombomere 2 bifurcate to form a short ascending branch, ending at the rhombomere 1/2 boundary ( Figure 2A), and a long descending branch to the upper cervical levels of the spinal cord ^5^. The dorso-ventral pattern reflects the initial inverted mandibular-maxillary-ophthalmic projection ( Figure 2A,B), whereas the antero-posterior facial fields covered by each trigeminal branch are mapped lateral (posterior) to medial (anterior ^5, 103^). As fibers extend along the hindbrain, second-order trigeminal neurons differentiate and may provide instruction to form a secondary axis along the lateral-medial plane ^5^. These features are particularly obvious in mutant mice with a doubling of the whisker-related barrel field ^102^. How the whisker afferents generate the respective “barrelettes” in the brainstem ^113^ is not yet understood in molecular detail ^5, 102^, but it is clear that activity mediated by *N*-methyl- d-aspartate (NMDA) receptors sharpens the map ^5, 103^.

**Table 1.** Timing of mouse sensory neurogenesis and map projection.

Sense	Sensory neuron “birthdate”	Sensory cell “birthdate”	Second-order neuronal “birthdate”	Afferents reach central target	Afferents reach peripheral target	References
Olfaction	OSN, continuous	OSN, continuous	E11-postnatal	Continuous	NA	116
Vision	E10.5–E13 central- peripheral	E11–15	E15–P0	E16–P0	NA	75
Somato- sensation	E8.25–9	NA, mostly free nerve endings	E10.5–15.5	E9.5–10	E12.5–15.5	5, 98
Balance	E9.5–E13.5	E10.5 postnatal	E9.5–15.5	E10.5	E10.5–E18.5	22, 117– 119
Hearing	E10.5–12.5 base-apex	E12.5–14.5 apex-base	E10.5–14.5	E12.5–14.5	E14.5–19.5	57, 58, 117, 120, 121
Taste	E8.5–10.5	Continuous	E10.5–14.5	E10.5	E13.5–14.5	64, 122, 117

Cell cycle exit gradients are clearly documented only in the retina (central to peripheral progression) and hearing (base to apex for spiral ganglion neurons, apex to base for hair cells, and high frequency to low frequency in anterior cochlear nuclei). NA, not applicable; OSN, olfactory sensory neuron that is also the sensory cell.

### Extirpation and transplantation of whiskers

Physical manipulation of the whiskers plays a role in the maturation of the “barrelettes” in the brainstem as well as cortical barrels ^101^. Transplantation of supernumerary whiskers causes formation of barrelettes in the trigeminal nucleus and barrel fields in the cortex, whereas blockade of activity prevents barrelette formation ^103^ without disrupting overall sensory projection patterns. How relative activity results in gene expression and altered cortical map configuration in the somatosensory system is currently being investigated ^106^.

## Vestibular maps for linear and angular acceleration detection

### Adult organization

Vestibular afferents to different end organs originate from overlapping populations of vestibular neurons within the vestibular ganglion ^114, 115^. Central projections from distinct end organs show that the two types of vestibular receptors—the canals for angular acceleration and the otoconia bearing linear acceleration organs—have both discrete and overlapping projections ^21, 123^, possibly reflecting that all angular acceleration prompts additional linear stimulation. Each of the segregated and common signals is related to rhombomere-specific nuclei with different outputs ^17, 63^. An added complexity, shared with the lateral line system of mechanosensors, is the opposing polarity of hair cells in linear but not angular acceleration sensors ^124, 125^.

### Development

Beyond descriptive analysis of development of central projections ^22^, no molecular analysis exists that could explain the partial and incomplete segregation of vestibular sensory neurons projecting to different end organs and the partially segregated and partially overlapping central projection. Afferents innervating hair cells with different polarities project centrally to different rostro-caudal targets, such as the cerebellum and caudal hindbrain ^126^. *Nrp2* plays a role in regulating bifurcation ^127^, but how a lack of bifurcation translates into a differential pattern of central and peripheral targets has not been revealed. Neural crest–derived Schwann cells provide some peripheral guidance ^128^ but seem to have no effect on central projections ^129^. Neither developing targets nor neurotrophic support from targets is needed to guide growing vestibular afferents to the correct ear organ ^130, 131^, but stop signals are needed to confine growing peripheral fibers to specific sensory organs ^132^. In the related system of lateral line mechanosensors, cell cycle exit of sensory neurons defines their central target, distinguishing between primary sensory cells connecting to the Mauthner cell and secondary sensory cells that lack such connections ^124^. How much of this development in fish plays a role in vestibular development in mammals remains speculative ^125^. More data on possible *ErbB* ^128, 133^ and *Eph* ^134, 135^ involvement in vestibular afferent ordering are warranted.

### Ear manipulations

Transplantation of developing ears ^136^ has established that guidance cues are highly conserved between vertebrates ^136, 137^. The ability to form functional connections with the hindbrain does not depend on the entry point of vestibular ganglion processes into the hindbrain. Functional rerouting to the vestibular nucleus of afferents from transplanted ears that entered into the spinal cord has been demonstrated ^138^. Transplantation and rotation of a third ear to generate non-matching stimulation relative to the native ear’s sensory epithelia result in “vestibular dominance columns” ^139^. These “vestibular dominance columns” may reflect a compromise between molecular guidance cues and their activity-related refinement that was first identified in the visual system ^33, 90^. More information on molecular and activity-mediated vestibular projection ordering is needed to guide restoration of vestibular function through neuronal transplantation to prevent falls of the growing number of seniors with vestibular neurosensory loss ^140^.

## Tonotopic map

### Adult organization

The auditory system segregates sounds of high to low frequencies along the base-to-apex length of the cochlea and projects this unidimensional frequency information via topographically restricted spiral ganglion neurons to discrete isofrequency bands within the cochlear nucleus complex ^9^, generating a single inner hair cell–to–projection band topology ( Figure 1C and Figure 2A,B). Second-order neurons project an isofrequency map onto third-order neurons ^141^ that use time and intensity differences to extract sound direction by comparing the identical frequency of the two ears ^16^ to generate a sound space map ^15^. Although cortical neurons can be excited by specific frequencies, the granularity and response properties of cortical neurons differ from those of brainstem neurons ^10^. The idea that the cortical tonotopic map is continuous at the microscale was recently questioned by using more sophisticated techniques: adjacent cortical response properties vary by up to three octaves, indicating a discontinuous microscale frequency map ^104^.

### Development

Of all maps, the cochleotopic map is the simplest in terms of projecting just one dimension ( Figure 1C), the linear arrangement of spiral ganglion neurons onto a matching linear projection in the cochlear nuclei ^55^. Despite this apparent simplicity relative to olfactory and optic maps, surprisingly little is known about the molecular basis of this primary map formation ^56^. Spiral ganglion neurons exit the cell cycle in a base-to-apex progression ^120, 57^ and project to their central targets within 48 hours after exiting the cell cycle ^121^ in an orderly arrangement of afferent fibers within the cochlear nerve ^56^. A sequence of transcription factors defines the neuronal precursors and their development ^142, 143^. Evidence on two of these transcription factors— *Neurod1* and *Gata3*—suggests their involvement in both peripheral and central process navigation by expressing yet-to-be-determined downstream factors in developing spiral ganglion neurons ^56, 144, 145^. How exactly these transcription factors regulate the essential interactions with Schwann cells to keep spiral ganglion neurons within the right position ^129^ or with various substrate information to navigate to distinct types of hair cells ^146, 147^ remains to be shown. For the first time in any primary sensory map, mouse mutants now exist with molecularly induced peripheral and central misguidance that cannot be corrected for by near-normal auditory activity ^56^. Consistent with developmental data in the somatosensory ^5^ and olfactory ^53^ system, neither peripheral nor central target cells are needed to develop an orderly projection ^148^ and partial loss of central targets has no obvious effect on the primary central segregation of spiral ganglion afferents ^149^. Primary afferents give rise to secondary branches to project a refined topographic map along the cochlear nuclei. Likely candidates for the molecular guidance of organized second-order fiber projection are Wnts released from the rhombic lip ^150^. Defects in mapping are prominent in mice mutant for *Prickle1*, a downstream effector of the *Wnt/Fzd* pathway ^151^. Furthermore, *Neurod1* is known to regulate *Fzd* receptors ^152^. In analogy to the retino-midbrain projection ^84^, *Wnts* may generate a gradient (or gradients) within which spiral ganglion afferents orient using a combination of *Fzd* and *Ryk*, both regulated by *Neurod1* ^152^. Other factors with limited effects are *Hox* genes, *Nrp2* and *Eph/ephrins* ^134, 56, 153^ and possibly neuropilins and semaphorins ^132, 147^.

### Experimental manipulations

Auditory refinement has been investigated by sound manipulation and surgical or molecular deletion of some parts of the adult or developing cochlea. Surviving spiral ganglion neurons remap remaining central afferents after either neurotrophin-mediated deletion ^154^ or various lesions of the auditory periphery ^155– 157^. This plasticity of the auditory system ^158, 159^ is likely governed by the Hebbian principle ^28^. Further studies on the recently described primary tonotopy-disrupted viable mice ^56^ could shed light onto limitations of such plastic reorganization. Such information is required for replacements of spiral ganglion neurons to improve hearing in the elderly with sound-induced neuropathy ^160^ or to improve cochlear implants ^140^ or replace ears ^137^.

## Primary taste maps challenge past taste concepts

### Adult organization

Many medical textbooks claim that different tastants are perceived by different taste buds and projected to distinct rostro-caudal subdivisions of the solitary tract ^24^. Further, it was thought that distinct information was gathered by different taste buds (fungiform, foliate, circumvallate papilla, and pharyngeal taste buds) and these tastants were carried by a separate cranial nerve innervating the different taste buds ( Figure 2A,B). Recent findings have radically changed this belief. A taste bud consists of 50 to 100 taste receptor cells ^36^, and all taste buds perceive all five tastants (sweet, sour, bitter, salty, and umami), each binding to a molecularly distinct receptor ^48, 161^. The graded taste information ^23^ is projected via three cranial nerves (VII, IX, X; Figure 2) to a dorso-ventral and rostro-caudal overlapping afferent distribution in the solitary tract that retains a rough orotopic organization ^26, 36, 162^. Highly conserved second-order neurons ^163^ project taste information to be combined with tongue-related somatosensation and olfaction into an integrated experience related to food intake ^1, 36^.

### Development

Taste neurons are generated by epibranchial placodes using unique sets of transcription factors ^164^. Peripheral processes of taste neurons are not needed for mammalian taste bud induction ^64, 165^ but rather for maintenance of taste buds ^166, 167^. How taste afferents navigate to reach the right peripheral target to interact with the developing taste buds is unclear but is apparently not dependent on the neurotrophin Bdnf ^168, 169^. Autonomy of central afferent navigation is achieved in mice mutants that owing to null mutation for *Tlx3* have no solitary nucleus development, but taste afferents seem to innervate adjacent nuclei in the absence of their specific target neurons ^170^. The expression of the solitary nucleus specifying transcription factor *Tlx3* is directed by *BMP* gradients ^111^. Owing to the early death of these mutants, it is unclear how long afferents can be maintained in the absence of their central target. Notably, taste ganglion neurons express neurotrophins to be self-supporting in the absence of a peripheral or central target ^168^. Because all taste buds perceive all tastants with various thresholds ^23^, it remains unclear what specific information the rough orotopic projection of afferents extracts and how the differential activity of each taste bud to various concentrations of tastants ^23^ can be used to sharpen the taste map. Clearly, the orotopic organization is lost in higher-order projections, making the need of the orotopic primary map even more fuzzy ^26^.

### Experimental manipulations

Crafting of tongues to foreign areas such as orbit and liver has long established the independence of taste bud development ^171, 172^. More experimental data are needed on the molecular guidance of taste afferents, the functional significance of orotopic organization, inter-solitary nucleus connections ^25, 173^, and higher-order interactions ^174^. Importantly, no data exist showing how the orotopic projection develops in the absence of taste buds ^165^ and where afferents end long term in the absence of a central target ^170^. Such information will be crucial to establish proper taste after complex orofacial surgery related to cancer or complex head trauma ^175, 167^.

## Overview of brainstem maps

Olfactory and retinotopic maps differ from brainstem maps as the former either involve the only mammalian sensory neuron/cell (the OSN) with its own axon that is continuously replaced or deal with a region of the brain transformed into the retina, generating the “optic nerve” out of an intracerebral tract. In addition, both of the above-described maps provide a point-to-point connection that either projects one surface (the retina) onto another surface (the midbrain ^8^) in two dimensions or ensures that a given odor binding to distributed OSNs converges on the same glomerulus (olfactory map ^33^). No such point-to-point map is obvious in the hindbrain ( Figure 1C and Figure 2A) where a given peripheral connection (such as a specific area of the facial skin or the cochlea) is innervated by a neuron residing in a given ganglion with a distinct molecular (Neurog1 versus Neurog2 ^177, 122^) and developmental (epibranchial placode, otic placode, trigeminal placode, and neural crest ^46, 73, 164^) origin. Instead of a point-to-point connection, each of the hindbrain targeting sensory neurons forms an extended longitudinal track along the alar plate of the hindbrain ( Figure 1C and Figure 2A). As a first approximation, the hindbrain alar plate can be regarded as a highly transformed part of the spinal cord that has developed rhombomere-specific nuclei, which receive hindbrain-specific innervation ^109, 63, 178, 179^. Within each of the longitudinal hindbrain tracks, rhombomere-specific nuclei can be identified ^5, 17, 178, 179– 182^, and each has its own higher-order projection. How the well-known cortical maps such as for somatosensation ^5, 6^ are exactly derived from the organizational principle of primary afferents ^102, 103^ is only in the case of the auditory and somatosensory system partially clarified ^5, 10, 11^. An emerging principle of hindbrain and visual map formation, likely not shared with the olfactory map because of its continuous replacement, is the role of cell cycle exit.

### Cell cycle exit influences topology

Developmental features that play no role in olfactory or only a modulatory role in the retinotopic map formation ^75^, such as timing of cell cycle exit and axonal projection, seem to play an underexplored part in overall brainstem map formation ^65, 183^. Neurons of the alar plate and cranial ganglia have distinct, partially overlapping cell cycle exits ^184– 117^. Migration within the alar plate, as in the spinal cord ^179^, suggests that more sophisticated pulse-chase experiments with modern EdU/BrdU double labeling are needed to resolve temporal maps. Indeed, a very recent article showed that the anterior parts of the cochlear nucleus complex show a coordinated cell cycle exit matching that of spiral ganglion neurons ^58^. The temporal progression of spiral ganglion cell cycle exit ^57, 120^ and progressive development of spiral ganglion neurons and their central projections ^121^ imply a birth-dating bias toward map formation ( Figure 1C and Figure 2B). In addition to “birth-dating” map of secondary neurons of the alar plate, the cell cycle exit varies among peripheral neurons ( Table 1), most obviously in the auditory system ^57, 120^. The epibranchial derived neurons innervating the taste buds of the tongue project early in development to the solitary tract ( Table 1), long recognized as the first tract to form in the mammalian hindbrain ^63, 170^. First-born and projecting neurons of a given ganglion form the most ventral projection in the alar plate and within a given alar plate nucleus ^65, 183^. In amphibians and fish, afferents of different senses developing at different times project in a ventral-to-dorsal progression ( Figure 2B) to the hindbrain ^65, 183^ and form distinct aspects of some lateral line sensory maps ^124^. Beyond the birth-date related primary afferent fiber organization, the formation of alar plate nuclei and side branches of primary afferents makes it difficult to extract primary map formation and to derive general organizational principles ^5^ in mammals without more refined analysis as recently conducted in the visual system ^75^ and auditory system ^58^. In addition to cell cycle exit of alar plate and sensory neurons, there is a rostro-caudal progression in maturation leading to a rhombomere-specific second-order neuron cell cycle exit, matching the arrival and formation of secondary branches of primary sensory afferents ^17, 103^. These data suggest that more refined analysis of temporal progression of molecular guidance cues is warranted for the brainstem and visual projection development.

## Summary

Primary sensory maps mirror the unique properties of a given sensory modality. Maps can reflect (a) local receptor density and activity (somatosensory), (b) convergence of distributed receptors (olfactory), (c) continuous one-dimensional (tonotopic) or (d) 2D (retinotopic and somatotopic) maps, or (e) convergence and segregation of information gathered by distinct sensory organs (vestibulotopic and orotopic) maps. An emerging principle of several maps is the role of cell cycle exit that allows distinct inputs to interact specifically with matching cell cycle–exited second-order neurons. This is particularly obvious in the temporal progression of afferent projections from various sensory systems in amphibians and bony fish, the temporal and maturational progression of spiral ganglion, and cochlear nucleus cell cycle exit, and it plays a role in RGN type specifications. Afferent fiber sorting prior to the target is an obvious common feature in all sensory systems and may reflect both fiber–fiber molecular interactions and cell cycle exit. As a consequence of fiber sorting, a crude topology of processes arises before a target is innervated and even in the absence of a target as demonstrated in the olfactory and auditory systems. Once the specific map has been established by various non-activity-related means, a common feature is that activity sharpens the map. To the best of our knowledge, there is only one developing sensory system currently known where a single deletion in a viable mutant results in near-random distribution of peripheral and central processes that cannot be corrected by physiological activity. Such mutants can test the limits of activity-mediated refinement of distorted primary maps.

Combinations of classic embryologic manipulations, such as transplantations, rotations, or partial deletions, have been extremely helpful to formulate basic principles such as the chemoaffinity theory. Whereas the topographical information coded in such diffusible gradients may be uniform across all sensory maps, the molecular nature of specific guidance cues used certainly is not. However, it is noteworthy that several maps have a dorso-ventral axis that could reflect known countergradients of diffusible molecules needed to define different dorso-ventral nuclei, such as *Bmp4*, *Wnt3*, and *Shh*. Going forward, combining heterochronic and heterotopic transplantations with molecular perturbation of map formation and with the evaluation of the role of activity to sharpen such distorted maps will reveal how best to use such information to enhance sensory organ replacements for functional recovery to cure anosmia, blindness, vestibular, and auditory dysfunction. Whole face or tongue transplants could also benefit from an understanding of such detailed map formation. Clearly, cortical maps will plastically respond to peripheral manipulations, but meaningful integration of various sensory information requires that each primary map be appropriately organized to allow a multisensory cortical or subcortical integration of relevant information extracted out of primary maps.

## Abbreviations

2D, two-dimensional; bHLH, basic helix-loop-helix; MesV, mesencephalic trigeminal nucleus; Nrp, natriuretic peptide receptor; OB, olfactory bulb; OR, odorant receptor; OSN, olfactory sensory neuron; RGN, retinal ganglion neuron
